# The Research Gap in Non-tuberculous Mycobacterium (NTM) and Reusable Medical Devices

**DOI:** 10.3389/fpubh.2020.00399

**Published:** 2020-08-20

**Authors:** Jon W. Weeks, Katharine Segars, Suvajyoti Guha

**Affiliations:** Center for Devices and Radiological Health, U. S. Food and Drug Administration, Silver Spring, MD, United States

**Keywords:** non-tuberculosis mycobacterium, slow growing mycobacteria, rapid growing mycobacteria, aerosolization, disinfection

## Non-Tuberculous Mycobacteria and Reusable Medical Devices

Patient infections with Non-tuberculous Mycobacterium (NTM) have been attributed to some reusable medical devices (1, 2), such as heater cooler devices (3–5), dental unit waterlines (6), bronchoscopes (7), and automated endoscope reprocessors (8, 9). Such incidents can be related to insufficient reprocessing or growth of resistant organisms. For example, NTM infections can arise from patient exposure to contaminated water from established biofilms in water systems, and in some cases, aerosolization of the contaminated water (10). These medical devices are regulated by the U.S. Food and Drug Administration (FDA) and the Agency seeks to better understand the mechanism by which devices can transmit NTM. Some NTM-specific challenges include potentially years-long incubation period to clinical infection, subsequent difficulty identifying the bacteria to the species-level and extended duration of treatment (11).

While general awareness around clinically relevant, rapid- and slow-growing NTM and infections appears to have increased (11), a literature review of Mycobacterium research reveals the trend of testing rapid-growing *Mycobacterium spp*. as surrogates and extrapolating data to their slow-growing counterparts (12–14). However, with more than 150 known NTM species (15), questions about the applicability of surrogate data have been voiced for decades (11, 13). Comparative analyses between slow- and rapid-growing NTM tend to be limited and in some cases, have demonstrated notable variation, as well as intra-species differences (13, 14, 16, 17). This raises questions about the applicability of using one species of NTM (herein referred to as surrogate NTM) in place of another for medical device testing. The topic of appropriate surrogates can be considered in many ways. However, in the context of this manuscript we will discuss the applicability of NTM surrogates for testing intermediate- and high-level disinfection^1^ of, and aerosolization from, reusable medical devices in the context of specific NTM outbreaks. Note that this manuscript does not address or suggests any changes in well-established disinfection validation methods that are routinely used for medical device testing.

Fundamental NTM research will be useful to advance medical device testing and support public health and safety. With this opinion piece, we highlight three areas of research, namely morphological characteristics, susceptibility to disinfectants, and aerosolization potential (if applicable based on device design and history of infection), that will enable the medical and research communities to identify commonalities and differences amongst NTM species. Further research into these areas will help inform which types of testing can mitigate risk of infections and prevent NTM outbreaks.

## Morphological Characteristics

Patient infections by exposure to NTM contaminated medical devices can occur through various mechanisms, including transmission through water or air. Morphology influences bacterial viability in these environments. *Mycobacterium* spp. exhibit different characteristics with variations in size (17), structure and shape, which can be impacted by the selected growth media (19). Further evidence suggests that some NTM species can alternate between smooth and rough colony morphotypes (20, 21). Yet it remains unclear whether these phenotypic differences alter adherence on medical devices, the propensity to aerosolize from solution and susceptibility to disinfectants.

Medical device colonization and subsequent biofilm formation could influence NTM presence in healthcare environments, with potential patient exposure through water and/or aerosols. Sousa et al. (22) showed differences between the rate of biofilm formation among three NTM species; *M. smegmatis, M. fortuitum*, and *M. chelonae*. If biofilm formation varies among NTM species, it is not clear that using a surrogate to evaluate biofilm removal from contaminated medical devices applies to all NTM, in particular those associated with public health concerns.

Ambiguity regarding NTM growth patterns has been further reinforced through recent findings. Vijay et al. (17) conducted studies with *M. smegmatis* and determined that this species may encompass “sub populations” with varied characteristics, such as cell size. Because cell size can influence bacterial aerosolization, size variability among species raises questions about extrapolating aerosolization data from one species to another and whether surrogate testing is relevant to demonstrate absence of aerosolization from medical devices.

## Disinfection and NTM Resistance

As with variability of morphological characteristics among NTM species, similar considerations apply for variability in disinfectant susceptibility (11). We reviewed current literature to investigate information regarding NTM susceptibility to common disinfectants (summarized in Table 1). The cited studies evaluated disinfectants in two categories; water treatment to control bacteria and the microbicidal process of disinfection. While the literature shows trends in disinfectant susceptibilities of *Mycobacterium* spp., in some cases different studies demonstrated dissimilar relative susceptibility amongst species (25, 26). This may be attributable to test conditions.

**Table 1:** Summary of relative susceptibility of NTM to chemicals used for disinfection.

**Disinfectant chemicals**	**Relative susceptibility amongst mycobacterium species**	**References**
Chlorine releasing compounds (including sodium hypochlorite and TC-101)	*M. fortuitum* and *M. chelonae* are more resistant than *M. gordonae* and *M. aurum*. *M. chelonei* subspecies *abscessus* is more resistant than *M. tuberculosi*s.	(13, 23)
Iodophor	*M. chelonei subspecies abscessus* and *M. tuberculosis* have similar susceptibility.	(23)
Phenol	*M. chelonae* and *M. terrae* are more resistant than *M. bovis*.	(14)
Silver nanoparticles	*M. smegmatis* is highly susceptible to silver nanoparticles (AgNP) compared to *M. avium* and *M. marinum*.	(24)
Glutaraldehyde	*M. tuberculosis, M. terrae, M. bovis, M. avium, M. abscessus*, and *M. chelonae* have similar susceptibility. However, glutaraldehyde-resistant clinical isolates have been identified for *M. abscessus* and *M. chelonae*. In a study of nine *Mycobacterium spp*., *M. smegmatis*, and *M. marinum* are highly susceptible to glutaraldehyde solutions; *M. avium, M. kansasii*, and *M. scrofulaceum* showed intermediate susceptibility; and *M. tuberculosis, M. bovis*, and *M. intracellular* were resistant, requiring higher concentrations and longer contact times to achieve reduction in counts.	(23, 25, 26)
Alcohol	*M. chelonei subspecies abscessus* and *M. tuberculosis* have similar susceptibility to alcohol. Resistance to glutaraldehyde does not alter resistance of *M. abscessus* to 15% isopropanol.	(23, 25)
Hydrogen peroxide	*M. tuberculosis, M. terrae, M. bovis, M. avium, M. abscessus*, and *M. chelonae* have similar susceptibility.	(25)
*Ortho*-phthalaldehyde (OPA)	Glutaraldehyde-resistant strains of *M. abscessus* and *M. chelonae* are similarly susceptible to OPA as glutaraldehyde-sensitive strains. *M. terrae* is more resistant than *M. chelonea* and *M. bovis*.	(14, 25)
Peracetic acid	*M. tuberculosis, M. terrae, M. bovis, M. avium, M. abscessus*, and *M. chelonae* have similar susceptibility.	(23, 25)
Quaternary ammonium compounds	*M. chelonae* and *M. abscessus* are more resistant than *M. smegmatis*. *M. terrae* is more resistant than *M. bovis*.	(25, 27)

Water is treated with chlorine or chloramine disinfectants to control bioburden in municipal water (16, 28) and improve water quality. Many NTM species are significantly more resistant to chlorine than *Escherichia coli*; for example, *M. avium* is more than 500-fold more resistant than *E. coli* to chlorine and is even more resistant when grown in water (11, 28–31). This suggests that water quality treatment may contribute to the selection and propagation of NTM by removing competitor organisms from the water supply (28–31). While it is important to control the bacterial load within the water, the effectiveness of various disinfection methods used for water treatment should be assessed against NTM to mitigate NTM proliferation within a water source.

To confirm that reusable medical devices can be intermediate- or high-level disinfected, studies typically demonstrate effective reduction of an appropriate *Mycobacterium* spp. (18). For general disinfection studies, *M. terrae* is sometimes used as a test organism, however, it is not necessarily regarded as the gold standard test organism and may not represent emergent NTM strains. Therefore, in emergent NTM outbreaks, additional scientific literature concerning comparative resistance would provide helpful information about strains used for validation testing. Literature indicates that *M. smegmatis*, a rapid-growing *Mycobacterium* spp., is often less resistant to disinfectants (13, 24, 26, 27). Because NTM vary in their susceptibility to some disinfectants, it is unclear whether testing medical device disinfection with *M. smegmatis* as a surrogate is applicable to clinically relevant NTM species (11).

Comparative analyses have been performed between some *Mycobacterium* spp. for certain disinfectants [reviewed in Table 1; (11, 13, 14, 23–27, 29–31)]. These analyses have been limited in the number of organisms or pertain to specific types of disinfectants. Burgess et al. (25) performed a comparative analysis between multiple *Mycobacterium* spp. (including rapid- and slow-growing NTM) to several clinically utilized disinfectants. More comprehensive studies like these would be beneficial to assess whether a specific strain is representative of most *Mycobacterium* spp. to a broad range of disinfectants. This information may inform disinfection validation methods and conditions for reusable medical devices linked to emergent NTM outbreaks.

## Aerosolization

The potential for an NTM to aerosolize likely depends on several factors including: cell size and morphology (32, 33), the hydrophobicity of the bacterial cell wall (34), and the mode of transfer. To date, studies characterizing these factors are rare. Therefore, this section highlights different areas of research that will help establish similarities and differences between the aerosolization potential of slow- and rapid-growing NTM.

In the context of size, the following factors are generally important for aerosols: diffusion, impaction, interception, gravitation and electrostatic interactions (35). Given that most bacteria are larger than 0.2 μm, diffusion may not be a significant factor. Impaction and electrostatic interactions are likely to be important when NTM moves through tight spaces (such as an air-filter). In open spaces, gravitational settling is expected to play the most important role for aerosols >1 μm size. Due to variability in the reported sizes of *Mycobacterium* spp. (17, 36), comparative analyses may elucidate the influence of species size on aerosol dispersion.

Studies to quantify the hydrophobicity and aerosolization potential of different NTM species compared to non-biological surrogates, such as polystyrene latex beads and silica beads will also be useful. Developing a one-to-one correlation between clinically relevant NTM species and beads may potentially support future studies using non-biological surrogates.

NTM transfer from water to air can happen in at least three ways: spraying (37), ultrasonication (32, 33, 38, 39), or bubbling (32, 40). For bacterial aerosolization studies, it is not clear if the mode of transfer influences post-aerosolization quantitation of viable, aerosolized test organisms. Stresses from aerosolization could rupture cells resulting in under reporting of microorganisms (41, 42). Having this information for clinically-relevant NTM species would help to inform the studies needed to evaluate aerosolization potential from medical devices.

While some physical and biological aerosolization analyses have been performed for *M. tuberculosis* (43), limited studies exist for other *Mycobacterium spp*. Both physical and biological assays have limitations. Physical analyzers (such as size spectrometers, impactors, and optical detectors) are non-specific and thus provide little information regarding the contents of aerosols. Conversely, biological assays (such as settle plates, impaction on solid agar, and impingement in solution) are more content-specific but rely on the viability of the organisms post-aerosolization and are prone to contamination. Based on the above limitations, a dual-approach utilizing both physical and biological detection may provide the best insights into NTM aerosolization from medical devices (e.g., heater-coolers). Additional studies comparing the accuracy of physical and biological assays may alleviate future need for both types of studies. Within biological assays, a study comparing collection method using *E. coli* and *Bacillus subtilis* (44) found that the collection efficiency can vary with the type of collection method and bacteria. Therefore, further analyses of collection methods for different NTM species using passive (such as settle plating) and active sampling [biosamplers or airport samplers; (45)] are needed. Time dependent survivability and recoverability of NTM should be determined to optimize biological aerosolization studies. Finally, a direct comparison of NTM aerosolization via the three modes mentioned (spraying, ultrasonication, and bubbling) would also be beneficial when considering risk of aerosolization from medical devices.

## Future Direction

The intent of introducing this discussion is not to provide regulatory guidance, but rather to elucidate areas where additional research may broaden the scientific awareness of NTM characteristics that are pertinent to patient safety. To summarize, such areas may include an in-depth characterization of the different NTM phenotypes (including size, shape, and disinfectant resistance), hydrophobicity, and assay-based NTM aerosol research. Assessing the relationships among these factors will help the scientific community better understand their influence on medical device contamination and subsequent patient infection. A recent study by Schreiber et al. (46), which evaluated the influence of water volume and media on the detection limit of *M. chimaera*, is a helpful step in that direction.

We look to the research community to support robust, optimized, and sensitive studies characterizing commonalities and differences among NTM species that will aid in determining appropriate surrogates and in better understanding aerosolization potential and susceptibility to disinfection. This information will assist public health representatives (i.e., clinicians, hospitals, industry, and regulators) in better understanding and mitigating risks associated with NTM infection.

## Author Contributions

SG and JW put forward the idea of a manuscript. KS, JW, and SG drafted the manuscript based on their respective expertise. All authors contributed to the article and approved the submitted version.

## Conflict of Interest

The authors declare that the research was conducted in the absence of any commercial or financial relationships that could be construed as a potential conflict of interest.

## Footnotes

^1^Intermediate-level disinfection is a microbicidal “process that kills viruses, mycobacteria, fungi, and vegetative bacteria, but not necessarily bacterial spores”; validated intermediate-level disinfection processes should demonstrate at least “6-log reduction of a mixed suspension of typical vegetative organisms, such as *Pseudomonas aeruginosa, Staphylococcus aureus, Escherichia coli*, and representatives of the Klebsiella-Enterobacter group, and a 3-log reduction of an appropriate *Mycobacterium* species” (18). High-level disinfection is a microbicidal process which has been demonstrated to kill “all microbial organisms but not necessarily large numbers of bacterial spores”; validated high-level disinfection processes should demonstrate at least “6-log reduction of an appropriate *Mycobacterium* species” (18).
